# It's the Recipient That Counts: Spending Money on Strong Social Ties Leads to Greater Happiness than Spending on Weak Social Ties

**DOI:** 10.1371/journal.pone.0017018

**Published:** 2011-02-10

**Authors:** Lara B. Aknin, Gillian M. Sandstrom, Elizabeth W. Dunn, Michael I. Norton

**Affiliations:** 1 Department of Psychology, University of British Columbia, Vancouver, British Columbia, Canada; 2 Marketing Unit, Harvard Business School, Boston, Massachusetts, United States of America; University of Maribor, Slovenia

## Abstract

Previous research has shown that spending money on others (*prosocial spending*) increases happiness. But, do the happiness gains depend on who the money is spent on? Sociologists have distinguished between *strong ties* with close friends and family and *weak ties*—relationships characterized by less frequent contact, lower emotional intensity, and limited intimacy. We randomly assigned participants to reflect on a time when they spent money on either a strong social tie or a weak social tie. Participants reported higher levels of positive affect after recalling a time they spent on a strong tie versus a weak tie. The level of intimacy in the relationship was more important than the type of relationship; there was no significant difference in positive affect after recalling spending money on a family member instead of a friend. These results add to the growing literature examining the factors that moderate the link between prosocial behaviour and happiness.

## Introduction

If you found a $10 bill in the pocket of an old jacket, what would be the best way to spend this money in order to maximize your happiness? Past research has shown that people are happier after spending money on others rather than spending on themselves [1]. Further, people benefit more from spending money on others when doing so provides them with the opportunity to spend time with another person (Aknin, Dunn, Sandstrom & Norton, submitted). However, no research has examined whether the happiness benefits of spending on someone else differ depending on who, specifically, the money is spent on. More broadly, little research has looked at the moderators that influence the affective boosts that people gain from performing acts of prosocial behaviour. Are people better off spending their newly found $10 bill buying coffee for their best friend, or for a friendly acquaintance from yoga class who they would like to know better?

There are many ways to classify the people and relationships in our lives. Perhaps one of the most obvious and straightforward ways is to group people by relationship type and use categories such as “family”, “friend” or “colleague”. While this relationship-based labeling system seems intuitive, it is somewhat limited because individuals within a particular category are not necessarily equivalent on dimensions such as closeness. For example, we would expect a relationship with a twin sister to be much different than a relationship with a rarely seen cousin, though both are family members. Another common way to classify social relationships is by level of intimacy [2]. Indeed, sociologists label relationships that involve less frequent contact, lower emotional intensity, and limited intimacy as *weak ties* [3]. These relationships are often considered in contrast to *strong ties* with close friends and family.

Considering this classification of our social relationships, one might wonder whether engaging in prosocial behaviour that involves strong versus weak social ties will lead to different happiness returns. While there is little research directly investigating this question, there is copious research examining the benefits of social relationships. A large body of evidence suggests that people enjoy interacting with strong ties; we are happier when we have satisfying relationships with close friends and family. A recent meta-analysis, which examined 22 studies with a range of well-being measures, touted the connection between well-being and social relationships as “…one of the most robust findings in the literature on well-being” [4]. However, researchers usually test for associations between well-being measures and prototypical strong tie measures such as marital satisfaction, or number of close friends; the effect of relationships with weak ties on well-being has seldom been explored.

While it may seem intuitive that strong ties have consequences for our well-being, research has also shown that our relationships with weak ties, and even strangers, can affect our happiness. Using a large-scale, longitudinal dataset, Fowler and Christakis [5] suggested that happiness spreads throughout social networks, extending up to three degrees of separation: a person becomes happier if their friend's friend's friend becomes happier, even if they don't know that person (see also [6]). Given that the presence of weak ties in our social network affects our happiness, it seems reasonable to hypothesize that prosocial behaviour directed towards weak social ties can provide similar affective benefits.

Extending these previous lines of research, the current study examines whether the happiness benefits that we garner from prosocial behaviour, and, in particular, spending money on others, differ depending on whether the target is a strong or weak social tie. Given the previous research demonstrating how important social relationships with close friends and family are for well-being, we hypothesized that participants would be happier after recalling spending money on a strong tie rather than a weak tie. However, given the positive effects that extend through weak ties in one's social network, it was also possible that participants would be equally happy after recalling spending money on weak ties.

## Methods

### Participants

Eighty individuals (68% female; *M_a_*_ge_  = 22.0, *SD* = 6.4) were approached in public places on the University of British Columbia campus and asked to participate in a study looking at how people spend money and how it affects their state of mind. One individual was removed from this sample because we suspected he did not take the study seriously; his response indicated that he recalled spending money on his “alter ego”. All participants provided written consent. This study was approved by the University of British Columbia's Behavioural Research Ethics Board (H06-80557).

### Procedure

Participants were randomly assigned to one of two spending recall conditions. They were asked to recall in as much detail as possible the last time they had spent approximately twenty dollars on either someone who they considered to be a strong social tie or someone they considered to be a weak social tie. Specifically, participants in the strong tie condition were asked to:

“Please think back to and describe as vividly and in as much detail as possible the last time you spent approximately twenty dollars ($20) on someone you are very close to (e.g., a good friend, close family member, romantic partner).”

Participants in the weak tie condition were asked to:

“Please think back to and describe as vividly and in as much detail as possible the last time you spent approximately twenty dollars ($20) on someone you are not very close to (e.g., an acquaintance, a co-worker, a classmate, a friend of a friend).”

After participants described their spending experience, they reported their current affect levels on the Positive and Negative Affect Schedule [7] (PANAS). This scale asks participants to report their current affect in response to 10 positive affect prompts (e.g., alert, interested, determined) and 10 negative affect prompts (e.g., guilty, scared, hostile), on a scale from “1 - very slightly or not at all” to “5 - extremely”. We added the adjective “happy” as an extra item on the PANAS inventory, since happiness was of primary interest to our study, and averaged the eleven positive affect items to form a measure of post-recall positive affect (α = .90).

After reporting their affect, participants provided further details about the spending experience, including how long ago the spending experience had occurred (1- in the last two days, 2- in the last week, 3- in the last month, 4- in the last year, 5- a long time ago).

## Results

We predicted that recalling a purchase made for a strong social tie would lead to higher levels of happiness than recalling a purchase made for a weak social tie. To investigate this question, we compared post-recall positive affect ratings for participants in the strong and weak social tie conditions using an independent samples t-test. There was a significant main effect of spending target, whereby participants randomly assigned to recall a purchase made for a strong tie reported feeling significantly more positive affect (*M* = 2.76, *SD* = .88) than participants assigned to recall a purchase made for a weak tie (*M* = 2.37, *SD* = .75), *t*(76)  = 2.09, *p*<.05. Thus, our prediction was supported: participants experienced greater well-being after reflecting upon a purchase made for a strong social tie.

We were also interested in how recently participants had spent money on strong or weak social ties. Given that the measure of the delay since spending used unequal time intervals (e.g., days vs. months), we used a non-parametric test. A Mann-Whitney test of the delay since spending indicated that participants spent significantly more recently on strong ties than on weak ties, *z* = -3.94, *p*<.001.

To ensure that the higher levels of positive affect reported in the strong social tie recall condition were not simply a result of more recent spending behaviour, we conducted an Analysis of Variance with delay since spending entered as a covariate. Supporting the robustness of the effect, participants who recalled a previous purchase made for a strong tie reported feeling more positive affect than participants who recalled a purchase made for a weak tie, even when controlling for the recency of the spending experience, *F*(1, 75)  = 3.91, *p* = .05.

We also investigated whether distinguishing between strong and weak social ties could account for the observed happiness differences better than distinguishing based on relationship type. When three individuals, blind to condition assignment, coded relationships as either family or friend (α = .97), there was substantial overlap between the relationship type classification (i.e., family vs. friends) and the intimacy level classification (i.e., strong vs. weak tie). Family members were more likely to be mentioned in the strong tie condition (vs. the weak tie condition) and friends were more likely to be mentioned in the weak tie condition, suggesting that these two classifications are related, χ^2^(1)  = 8.68, *p*<.005. Importantly, however, when both the family vs. friends and strong vs. weak tie classifications were entered into a regression, only the strong vs. weak tie distinction predicted differences in happiness levels following the spending recall (*β* = −.26, *p*<.05). These results suggest that level of intimacy is a more powerful predictor than the relationship type (*β* = .08, *ns*) when it comes to the happiness people reap from prosocial spending.

## Discussion

These data suggest that spending money on people we know well leads to higher levels of happiness than spending money on acquaintances. When participants were randomly assigned to recall a time they had spent money on either a strong or weak social tie, participants who recalled spending on a strong tie reported higher happiness afterward. As such, these findings suggest that to reap the greatest emotional reward from spending on someone else, one should direct their purchases to close others.

Consistent with this finding, research on reciprocal altruism and the evolution of cooperation demonstrates that people ultimately benefit from behaving generously and cooperatively toward individuals with whom they are likely to interact in the future [8]–[10]. Our results suggest that positive feelings arising from sharing one's resources with strong social ties may be one mechanism by which such behaviour was reinforced, and thus may serve an evolutionarily adaptive function.

The present findings also provide empirical support for the value of relying on participants' definition of what constitutes a strong or weak social tie. While it may seem intuitive to classify spending targets into categories based on relationship type – such that friends and family would be classified as “strong ties” – our data suggest that this distinction lacks the same predictive power as participants' self-categorization of the people in their lives as strong or weak ties. Future research on the emotional benefits of prosocial behaviour could benefit from considering the level of intimacy in a relationship rather than categorizing individuals by relationship type.

These findings should not be taken to suggest that people should avoid spending on weak social ties. Indeed, treating an acquaintance from yoga to a coffee after class might help to build a new strong tie. Thus, spending money on a weak social tie might help facilitate the development of new strong ties in the longer term.

This study is not without limitations. One simultaneous strength and shortcoming of this design is that participants were asked to recall a previous spending experience, rather than engage in a new spending behaviour for the purposes of this study. While this methodology may leave open questions of whether the same emotions would occur immediately after spending, this reminiscence-based methodology has been used successfully in previous research [11] and captures the kind of remembered utility that is an important component of the overall utility of experiences [12], [13].

Past research in our lab has repeatedly shown that people are happier when they use financial resources to benefit others rather than themselves [Aknin, Dunn, Sandstrom & Norton, submitted, [1], [14]]. Given that our aim in the current study was to answer the question of whether the happiness we gain from spending on others depends on who, specifically, the money is spent on, we did not include a condition in which participants were asked to recall spending on themselves.

Finally, this work adds to the growing body of empirical research suggesting how people might best spend money on others in order to reap emotional benefits and, more broadly, determining the conditions under which prosocial behaviour might lead to happiness. Knowing that individuals are happier after engaging in prosocial behaviour directed toward strong rather than weak social ties allows for simple and straight-forward applications.

Of course, further research should examine why engaging in prosocial behaviour directed towards strong social ties leads to greater happiness, how long the mood benefits last, and whether doing kind deeds for an acquaintance helps transform a shallow relationship into a deep friendship. The current results, however, shed novel insight into translating spending choices into happiness: the next time you find a few spare dollars in your pocket, you will be happiest if you treat your best friend.
